# Crystal structure of 4,6-di­amino-2-sulfanyl­idene-1,2-di­hydro­pyridine-3-carbo­nitrile

**DOI:** 10.1107/S1600536814018029

**Published:** 2014-08-09

**Authors:** Shaaban K. Mohamed, Mehmet Akkurt, Kuldip Singh, Bahgat R. M. Hussein, Mustafa R. Albayati

**Affiliations:** a Chemistry and Environmental Division, Manchester Metropolitan University, Manchester M1 5GD, England; b Chemistry Department, Faculty of Science, Minia University, 61519 El-Minia, Egypt; c Department of Physics, Faculty of Sciences, Erciyes University, 38039 Kayseri, Turkey; d Department of Chemistry, University of Leicester, Leicester, England; e Chemistry Department, Faculty of Science, Sohag University, 82524 Sohag, Egypt; f Kirkuk University, College of Science, Department of Chemistry, Kirkuk, Iraq

**Keywords:** crystal structure, polyfuntional pyridines, 3-cyano­pyridine-2(1*H*)-thio­nes, hydrogen bonding

## Abstract

The title compound, C_6_H_6_N_4_S, crystallizes with two independent mol­ecules, *A* and *B*, in the asymmetric unit. Both independent mol­ecules are almost planar [maximum deviations of 0.068 (6) Å in mol­ecule *A* and 0.079 (6) Å in mol­ecule *B*]. In the crystal, mol­ecules *A* and *B* are linked by N—H⋯S, N—H⋯N and C—H⋯S hydrogen bonds, forming a three-dimensional network.

## Related literature   

For the synthesis of polyfuntional pyridines, see: Attaby *et al.* (1995 ▶); Asadov *et al.* (2003 ▶). For various biological properties of pyridine scaffold compounds, see: Abdel-Rahman *et al.* (2002 ▶); Rao *et al.* (2006 ▶). For the synthesis of 3-cyano­pyridine-2(1*H*)-thio­nes, see: Fahmy & Mohareb (1986 ▶); Schmidt & Kubitzek (1960 ▶). For the use of 3-cyano­pyridine-2(1*H*)-thi­ones in the synthesis of bio-active compounds, see: Taylor *et al.* (1983 ▶); Gangiee *et al.* (1991 ▶); Amr *et al.* (2003 ▶); Abu-Shanab *et al.* (2002 ▶); Awad *et al.* (1962 ▶); El-Gaby (2004 ▶); Miky & Zahkoug (1997 ▶; Guerrera *et al.* (1993 ▶); Krauze *et al.* (1999 ▶). For a similar crystal structure, see: Eyduran *et al.* (2007 ▶). For the synthesis of the title compound, see: Abu-Shanab (1999 ▶). For standard bond-length data, see: Allen *et al.* (1987 ▶).
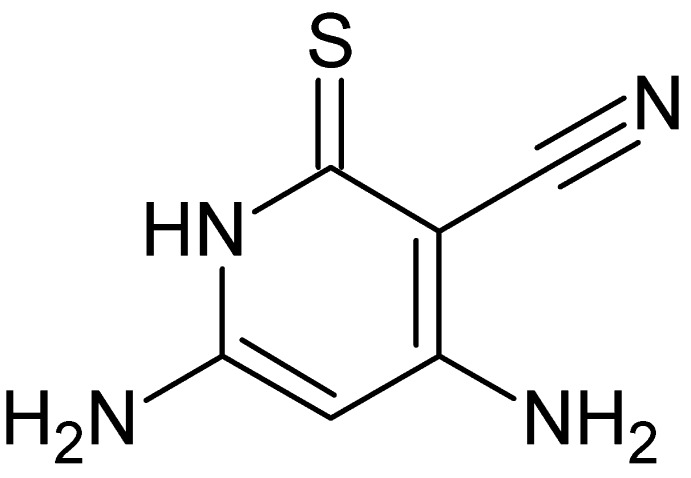


## Experimental   

### Crystal data  

C_6_H_6_N_4_S
*M* *_r_* = 166.21
Orthorhombic, 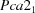

*a* = 26.252 (8) Å
*b* = 4.3670 (14) Å
*c* = 12.523 (4) Å
*V* = 1435.7 (8) Å^3^
*Z* = 8
Mo *K*α radiation
μ = 0.38 mm^−1^
*T* = 150 K
0.32 × 0.12 × 0.04 mm

### Data collection  

Bruker APEX 2000 CCD area-detector diffractometer
Absorption correction: multi-scan (*SADABS*: Bruker, 2011 ▶) *T*_min_ = 0.518, *T*_max_ = 0.928
11662 measured reflections
3412 independent reflections
2027 reflections with *I* > 2σ(*I*)
*R*_int_ = 0.137

### Refinement  

*R*[*F* ^2^ > 2σ(*F* ^2^)] = 0.064
*wR*(*F* ^2^) = 0.117
*S* = 0.87
3412 reflections
199 parameters
1 restraint
H-atom parameters constrained
Δρ_max_ = 0.42 e Å^−3^
Δρ_min_ = −0.34 e Å^−3^
Absolute structure: Flack (1983 ▶), 1573 Friedel pairs
Absolute structure parameter: 0.01 (13)

### 

Data collection: *SMART* (Bruker, 2011 ▶); cell refinement: *SAINT* (Bruker, 2011 ▶); data reduction: *SAINT*; program(s) used to solve structure: *SHELXS97* (Sheldrick, 2008 ▶); program(s) used to refine structure: *SHELXL97* (Sheldrick, 2008 ▶); molecular graphics: *SHELXTL* (Sheldrick, 2008 ▶); software used to prepare material for publication: *SHELXTL* and *PLATON* (Spek, 2009 ▶).

## Supplementary Material

Crystal structure: contains datablock(s) global, I. DOI: 10.1107/S1600536814018029/hg5399sup1.cif

Structure factors: contains datablock(s) I. DOI: 10.1107/S1600536814018029/hg5399Isup2.hkl

Supporting information file. DOI: 10.1107/S1600536814018029/hg5399Isup3.cml

**Fig. 1.**
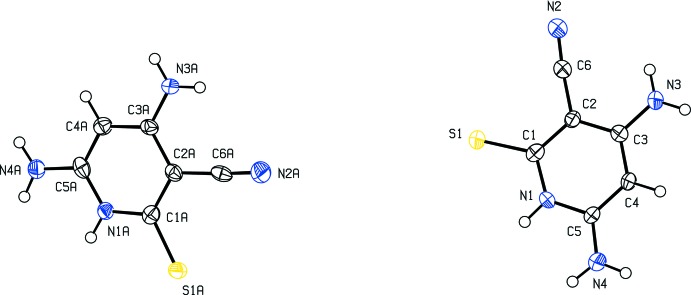
The title molecule showing the numbering scheme. Displacement ellipsoids are drawn at the 50% probability level.

**Fig. 2.**
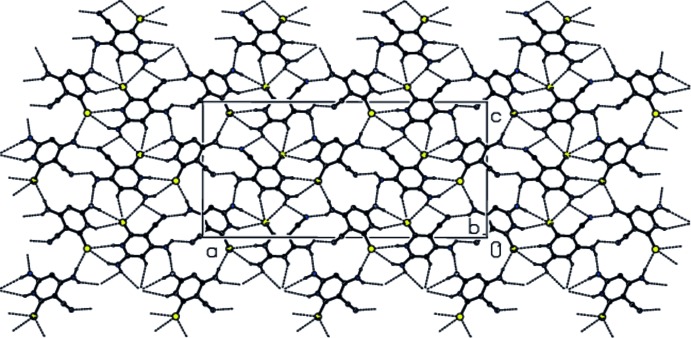
Packing viewed down the b axis showing the hydrogen bonding as dashed lines.

. DOI: 10.1107/S1600536814018029/hg5399fig1.tif
The title mol­ecule showing the numbering scheme. Displacement ellipsoids are drawn at the 50% probability level.

b . DOI: 10.1107/S1600536814018029/hg5399fig2.tif
Packing viewed down the *b* axis showing the hydrogen bonding as dashed lines.

CCDC reference: 1018166

Additional supporting information:  crystallographic information; 3D view; checkCIF report

## Figures and Tables

**Table 1:** Hydrogen-bond geometry (Å, °)

*D*—H⋯*A*	*D*—H	H⋯*A*	*D*⋯*A*	*D*—H⋯*A*
N1—H1⋯S1*A* ^i^	0.88	2.44	3.293 (5)	163
N1*A*—H1*A*⋯S1^ii^	0.88	2.80	3.579 (5)	149
N3—H31⋯N4*A* ^iii^	0.88	2.44	3.300 (8)	165
N3—H32⋯N2*A* ^iv^	0.88	2.39	3.077 (8)	135
N3*A*—H33⋯S1*A* ^ii^	0.88	2.53	3.392 (6)	168
N3*A*—H34⋯N2^v^	0.88	2.20	2.981 (8)	148
N4—H41⋯S1*A* ^i^	0.88	2.75	3.536 (6)	149
N4—H42⋯S1^vi^	0.88	2.63	3.424 (6)	151
N4—H42⋯N2^vi^	0.88	2.62	3.083 (9)	114
N4*A*—H44⋯S1^ii^	0.88	2.53	3.353 (6)	157
C4—H4⋯S1^vi^	0.95	2.74	3.551 (8)	143

Symmetry codes: (i) 
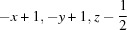
; (ii) 
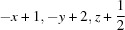
; (iii) 
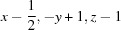
; (iv) 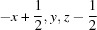; (v) 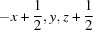; (vi) 
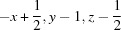
.

## supplementary crystallographic information

### S1. Comment

Polyfunctional pyridines are highly reactive reagents that have been used extensively in heterocyclic synthesis (Attaby *et al.*, 1995; Asadov *et al.*, 2003). 3-Cyano-pyridine-2(1*H*)-thiones compounds (Fahmy & Mohareb 1986; Schmidt & Kubitzek, 1960) have also gained considerable interest due to their importance as intermediates for the synthesis of the biologically active deazafolic acid and deaza amino protein ring system (Taylor *et al.*, 1983; Gangiee *et al.*, 1991). In addition, 3-Cyano-2(1*H*)-pyridinethiones and their related compounds were found to be very reactive substances for the synthesis of many different heterocyclic systems which exhibited biological activities such as antibacterial, pesticidal, antifungal, acaricidal and neurotropic activities (Amr *et al.*, 2003; Abu-Shanab *et al.*, 2002; Awad *et al.*, 1962; El-Gaby, 2004; Miky & Zahkoug, 1997; Guerrera *et al.*, 1993; Krauze *et al.*, 1999; Abdel-Rahman *et al.*, 2002; Rao *et al.*, 2006). In light of these observations we report in this study the synthesis and crystal structure of the title compound.

In the title compound (Fig. 1), the asymmetric unit contains two independent molecules (A and B). Molecules A and B both are almost planar (Fig. 3), with the maximum deviations of -0.068 (6) Å for N4 in molecule A and 0.079 (6) Å for N2A in molecule B. The bond lengths of molecules A and B are comparable to those of the previously published structures (Eyduran *et al.*, 2007; Allen *et al.*, 1987).

In the crystal, the N—H···S, N—H···N and C—H···S hydrogen bonds connect the molecules, forming a three dimensional network (Table 1, Fig. 2). In addition, C—H···π interactions and π-π stacking interactions are not observed.

### S2. Experimental

The title compound was prepared according to the reported method (Abu-Shanab, 1999). Crystals of the product were obtained in excellent yield (79%) and recrystallized from butanol to afford yellow needles (*M*.p. 583 K) in a sufficient quality for X-ray diffraction studies.

### S3. Refinement

H-atoms were placed in calculated positions (C—H = 0.95 and N—H = 0.88 Å and were included as riding contributions with isotropic displacement parameters 1.2 times those of the attached atoms.

### Crystal data

**Table:**

C_6_H_6_N_4_S	*F*(000) = 688
*M**_r_* = 166.21	*D*_x_ = 1.538 Mg m^−^^3^
Orthorhombic, *P**c**a*2_1_	Mo *K*α radiation, λ = 0.71073 Å
Hall symbol: P 2c -2ac	Cell parameters from 748 reflections
*a* = 26.252 (8) Å	θ = 2.3–23.4°
*b* = 4.3670 (14) Å	µ = 0.38 mm^−^^1^
*c* = 12.523 (4) Å	*T* = 150 K
*V* = 1435.7 (8) Å^3^	Needle, pale yellow
*Z* = 8	0.32 × 0.12 × 0.04 mm

### Data collection

**Table:**

Bruker APEX 2000 CCD area-detector diffractometer	3412 independent reflections
Radiation source: fine-focus sealed tube	2027 reflections with *I* > 2σ(*I*)
Graphite monochromator	*R*_int_ = 0.137
phi and ω scans	θ_max_ = 28.7°, θ_min_ = 1.6°
Absorption correction: multi-scan (*SADABS*: Bruker, 2011)	*h* = −34→33
*T*_min_ = 0.518, *T*_max_ = 0.928	*k* = −5→5
11662 measured reflections	*l* = −16→16

### Refinement

**Table:**

Refinement on *F*^2^	Hydrogen site location: inferred from neighbouring sites
Least-squares matrix: full	H-atom parameters constrained
*R*[*F*^2^ > 2σ(*F*^2^)] = 0.064	*w* = 1/[σ^2^(*F*_o_^2^) + (0.0282*P*)^2^] where *P* = (*F*_o_^2^ + 2*F*_c_^2^)/3
*wR*(*F*^2^) = 0.117	(Δ/σ)_max_ < 0.001
*S* = 0.87	Δρ_max_ = 0.42 e Å^−^^3^
3412 reflections	Δρ_min_ = −0.34 e Å^−^^3^
199 parameters	Absolute structure: Flack (1983), 1573 Friedel pairs
1 restraint	Absolute structure parameter: 0.01 (13)

### Special details

**Table:**

Geometry. Bond distances, angles *etc*. have been calculated using the rounded fractional coordinates. All su's are estimated from the variances of the (full) variance-covariance matrix. The cell e.s.d.'s are taken into account in the estimation of distances, angles and torsion angles
Refinement. Refinement on *F*^2^ for ALL reflections except those flagged by the user for potential systematic errors. Weighted *R*-factors *wR* and all goodnesses of fit *S* are based on *F*^2^, conventional *R*-factors *R* are based on *F*, with *F* set to zero for negative *F*^2^. The observed criterion of *F*^2^ > σ(*F*^2^) is used only for calculating -*R*-factor-obs *etc*. and is not relevant to the choice of reflections for refinement. *R*-factors based on *F*^2^ are statistically about twice as large as those based on *F*, and *R*-factors based on ALL data will be even larger.

### Fractional atomic coordinates and isotropic or equivalent isotropic displacement parameters (Å^2^)

**Table:**

	*x*	*y*	*z*	*U*_iso_*/*U*_eq_	
S1	0.27967 (6)	0.5692 (5)	0.10895 (15)	0.0332 (6)	
S1A	0.59399 (6)	0.7628 (4)	0.41599 (15)	0.0279 (5)	
N1	0.28114 (18)	0.2124 (13)	−0.0607 (4)	0.027 (2)	
N2	0.1365 (2)	0.4860 (15)	0.1504 (5)	0.039 (2)	
N3	0.1288 (2)	0.0258 (14)	−0.0735 (5)	0.037 (2)	
N4	0.2980 (2)	−0.1066 (15)	−0.2027 (5)	0.040 (2)	
C1	0.2513 (3)	0.3403 (15)	0.0166 (5)	0.026 (2)	
C2	0.1995 (2)	0.2719 (16)	0.0115 (5)	0.025 (2)	
C3	0.1797 (2)	0.0835 (15)	−0.0684 (5)	0.025 (2)	
C4	0.2122 (2)	−0.0471 (17)	−0.1416 (6)	0.030 (3)	
C5	0.2637 (2)	0.0167 (17)	−0.1372 (6)	0.027 (3)	
C6	0.1661 (2)	0.3946 (18)	0.0905 (6)	0.031 (3)	
N1A	0.59425 (19)	1.1254 (12)	0.5874 (4)	0.0230 (17)	
N2A	0.4628 (2)	0.4521 (15)	0.4637 (5)	0.038 (2)	
N3A	0.45527 (19)	0.8585 (13)	0.7032 (4)	0.0327 (19)	
N4A	0.6065 (2)	1.4593 (13)	0.7299 (4)	0.0277 (19)	
C1A	0.5677 (3)	0.9133 (16)	0.5295 (5)	0.025 (2)	
C2A	0.5201 (2)	0.8249 (16)	0.5670 (5)	0.023 (2)	
C3A	0.5005 (3)	0.9504 (16)	0.6643 (5)	0.022 (2)	
C4A	0.5300 (2)	1.1636 (15)	0.7195 (6)	0.026 (2)	
C5A	0.5760 (2)	1.2555 (16)	0.6797 (5)	0.026 (2)	
C6A	0.4896 (2)	0.6151 (16)	0.5089 (6)	0.025 (2)	
H1	0.31370	0.25940	−0.06120	0.0320*	
H4	0.19930	−0.18060	−0.19500	0.0360*	
H31	0.11670	−0.09500	−0.12370	0.0450*	
H32	0.10800	0.10930	−0.02670	0.0450*	
H41	0.33050	−0.06070	−0.19550	0.0480*	
H42	0.28820	−0.23400	−0.25310	0.0480*	
H1A	0.62450	1.18140	0.56440	0.0270*	
H4A	0.51800	1.24540	0.78520	0.0310*	
H33	0.44400	0.93300	0.76400	0.0400*	
H34	0.43690	0.72390	0.66770	0.0400*	
H43	0.59690	1.53930	0.79120	0.0330*	
H44	0.63580	1.51200	0.70130	0.0330*	

### Atomic displacement parameters (Å^2^)

**Table:**

	*U*^11^	*U*^22^	*U*^33^	*U*^12^	*U*^13^	*U*^23^
S1	0.0253 (9)	0.0437 (12)	0.0307 (10)	−0.0029 (9)	−0.0017 (8)	−0.0139 (10)
S1A	0.0223 (8)	0.0385 (10)	0.0229 (8)	0.0018 (9)	0.0020 (8)	0.0010 (9)
N1	0.016 (3)	0.037 (4)	0.027 (4)	−0.001 (3)	−0.001 (2)	−0.005 (3)
N2	0.026 (3)	0.054 (5)	0.037 (4)	0.002 (3)	−0.001 (3)	−0.011 (3)
N3	0.024 (3)	0.054 (4)	0.034 (4)	−0.004 (3)	−0.002 (3)	−0.020 (4)
N4	0.029 (3)	0.057 (5)	0.034 (4)	−0.009 (3)	0.004 (3)	−0.020 (3)
C1	0.031 (4)	0.025 (4)	0.021 (4)	0.003 (3)	0.000 (3)	−0.001 (3)
C2	0.023 (4)	0.031 (4)	0.021 (4)	0.005 (3)	−0.003 (3)	−0.006 (3)
C3	0.022 (3)	0.028 (4)	0.025 (4)	−0.002 (3)	0.001 (3)	−0.001 (4)
C4	0.025 (4)	0.034 (5)	0.030 (4)	−0.005 (3)	0.000 (3)	−0.017 (4)
C5	0.026 (4)	0.036 (5)	0.020 (4)	−0.003 (3)	0.001 (3)	−0.008 (3)
C6	0.023 (4)	0.040 (5)	0.031 (5)	0.001 (3)	−0.001 (3)	−0.007 (4)
N1A	0.022 (3)	0.027 (3)	0.020 (3)	−0.003 (3)	−0.001 (2)	0.002 (3)
N2A	0.035 (4)	0.042 (4)	0.036 (4)	−0.009 (3)	0.001 (3)	−0.003 (3)
N3A	0.021 (3)	0.049 (4)	0.028 (3)	−0.004 (3)	0.005 (3)	−0.012 (3)
N4A	0.026 (3)	0.032 (4)	0.025 (3)	−0.003 (3)	−0.002 (2)	0.000 (3)
C1A	0.028 (4)	0.023 (4)	0.023 (4)	0.004 (3)	−0.003 (3)	0.001 (3)
C2A	0.019 (3)	0.026 (4)	0.025 (4)	0.002 (3)	−0.002 (3)	0.004 (3)
C3A	0.018 (3)	0.026 (4)	0.023 (4)	0.006 (3)	0.000 (3)	0.003 (3)
C4A	0.027 (4)	0.025 (4)	0.026 (4)	0.002 (3)	0.001 (3)	−0.001 (3)
C5A	0.023 (4)	0.026 (4)	0.030 (4)	0.000 (3)	−0.008 (3)	0.009 (4)
C6A	0.020 (4)	0.024 (4)	0.032 (4)	0.006 (3)	0.005 (3)	0.007 (4)

### Geometric parameters (Å, º)

**Table:**

S1—C1	1.700 (7)	N1A—C5A	1.374 (8)
S1A—C1A	1.711 (7)	N1A—C1A	1.367 (9)
N1—C1	1.365 (9)	N2A—C6A	1.150 (9)
N1—C5	1.363 (9)	N3A—C3A	1.345 (9)
N2—C6	1.151 (9)	C4—H4	0.9500
N3—C3	1.361 (7)	N4A—C5A	1.352 (8)
N4—C5	1.332 (9)	C1A—C2A	1.390 (9)
C1—C2	1.394 (9)	N1A—H1A	0.8800
N1—H1	0.8800	C2A—C3A	1.432 (9)
C2—C3	1.396 (9)	C2A—C6A	1.418 (9)
C2—C6	1.426 (9)	C3A—C4A	1.394 (10)
C3—C4	1.376 (9)	N3A—H34	0.8800
N3—H31	0.8800	N3A—H33	0.8800
N3—H32	0.8800	C4A—C5A	1.367 (8)
N4—H41	0.8800	N4A—H43	0.8800
N4—H42	0.8800	N4A—H44	0.8800
C4—C5	1.382 (8)	C4A—H4A	0.9500
			
C1—N1—C5	124.2 (5)	C5—C4—H4	120.00
S1—C1—N1	118.1 (6)	S1A—C1A—N1A	119.7 (5)
S1—C1—C2	125.8 (5)	S1A—C1A—C2A	122.5 (5)
N1—C1—C2	116.1 (6)	N1A—C1A—C2A	117.9 (6)
C1—N1—H1	118.00	C1A—N1A—H1A	118.00
C5—N1—H1	118.00	C5A—N1A—H1A	118.00
C1—C2—C3	121.5 (6)	C1A—C2A—C3A	120.3 (6)
C1—C2—C6	119.2 (6)	C1A—C2A—C6A	120.9 (6)
C3—C2—C6	119.3 (5)	C3A—C2A—C6A	118.8 (6)
N3—C3—C2	120.6 (5)	N3A—C3A—C2A	120.8 (6)
C3—N3—H31	120.00	C3A—N3A—H33	120.00
C3—N3—H32	120.00	C3A—N3A—H34	120.00
H31—N3—H32	120.00	H33—N3A—H34	120.00
C2—C3—C4	119.4 (5)	C2A—C3A—C4A	118.6 (6)
N3—C3—C4	120.0 (6)	N3A—C3A—C4A	120.7 (6)
C5—N4—H42	120.00	C5A—N4A—H44	120.00
C5—N4—H41	120.00	C5A—N4A—H43	120.00
C3—C4—C5	119.8 (6)	C3A—C4A—C5A	120.4 (7)
H41—N4—H42	120.00	H43—N4A—H44	120.00
N1—C5—N4	117.3 (5)	N1A—C5A—N4A	117.2 (5)
N4—C5—C4	123.8 (7)	N4A—C5A—C4A	123.2 (6)
N1—C5—C4	118.9 (6)	N1A—C5A—C4A	119.6 (6)
N2—C6—C2	175.5 (7)	N2A—C6A—C2A	176.7 (6)
C1A—N1A—C5A	123.3 (5)	C3A—C4A—H4A	120.00
C3—C4—H4	120.00	C5A—C4A—H4A	120.00
			
C5—N1—C1—S1	177.7 (5)	C5A—N1A—C1A—S1A	−179.5 (5)
C5—N1—C1—C2	−3.0 (10)	C5A—N1A—C1A—C2A	−0.5 (10)
C1—N1—C5—C4	3.5 (10)	C1A—N1A—C5A—C4A	2.6 (9)
C1—N1—C5—N4	−175.4 (6)	C1A—N1A—C5A—N4A	179.1 (6)
S1—C1—C2—C6	−1.5 (10)	S1A—C1A—C2A—C6A	−3.5 (10)
S1—C1—C2—C3	179.3 (5)	S1A—C1A—C2A—C3A	178.0 (5)
N1—C1—C2—C3	0.0 (10)	N1A—C1A—C2A—C3A	−1.1 (10)
N1—C1—C2—C6	179.2 (6)	N1A—C1A—C2A—C6A	177.5 (6)
C1—C2—C3—N3	−178.3 (6)	C1A—C2A—C3A—N3A	−177.7 (6)
C6—C2—C3—C4	−176.9 (7)	C6A—C2A—C3A—C4A	−178.1 (6)
C1—C2—C3—C4	2.4 (10)	C1A—C2A—C3A—C4A	0.5 (10)
C6—C2—C3—N3	2.4 (10)	C6A—C2A—C3A—N3A	3.7 (10)
N3—C3—C4—C5	178.8 (7)	N3A—C3A—C4A—C5A	179.8 (6)
C2—C3—C4—C5	−1.9 (10)	C2A—C3A—C4A—C5A	1.6 (10)
C3—C4—C5—N4	177.9 (7)	C3A—C4A—C5A—N4A	−179.4 (6)
C3—C4—C5—N1	−0.9 (11)	C3A—C4A—C5A—N1A	−3.2 (10)

### Hydrogen-bond geometry (Å, º)

**Table:**

*D*—H···*A*	*D*—H	H···*A*	*D*···*A*	*D*—H···*A*
N1—H1···S1*A*^i^	0.88	2.44	3.293 (5)	163
N1*A*—H1*A*···S1^ii^	0.88	2.80	3.579 (5)	149
N3—H31···N4*A*^iii^	0.88	2.44	3.300 (8)	165
N3—H32···N2*A*^iv^	0.88	2.39	3.077 (8)	135
N3*A*—H33···S1*A*^ii^	0.88	2.53	3.392 (6)	168
N3*A*—H34···N2^v^	0.88	2.20	2.981 (8)	148
N4—H41···S1*A*^i^	0.88	2.75	3.536 (6)	149
N4—H42···S1^vi^	0.88	2.63	3.424 (6)	151
N4—H42···N2^vi^	0.88	2.62	3.083 (9)	114
N4*A*—H44···S1^ii^	0.88	2.53	3.353 (6)	157
C4—H4···S1^vi^	0.95	2.74	3.551 (8)	143

Symmetry codes: (i) −*x*+1, −*y*+1, *z*−1/2; (ii) −*x*+1, −*y*+2, *z*+1/2; (iii) *x*−1/2, −*y*+1, *z*−1; (iv) −*x*+1/2, *y*, *z*−1/2; (v) −*x*+1/2, *y*, *z*+1/2; (vi) −*x*+1/2, *y*−1, *z*−1/2.
