# Supplementary figures and images for: Crystal structure of 4,6-di­amino-2-sulfanyl­idene-1,2-di­hydro­pyridine-3-carbo­nitrile
Source: Acta Crystallogr Sect E Struct Rep Online. 2014 Aug 9;70(Pt 9):o993–4. doi: 10.1107/S1600536814018029 (PMC4186145; doi:10.1107/S1600536814018029)

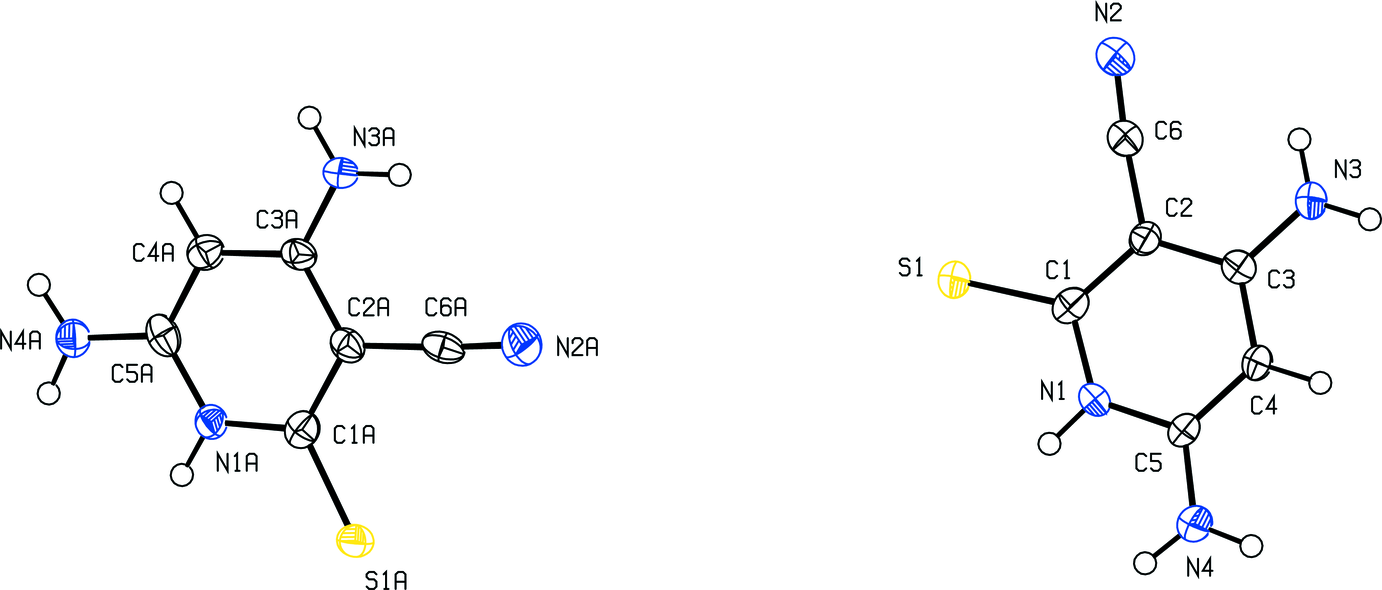

Supplement: Supplementary file 4 [file e-70-0o993-fig1.tif]

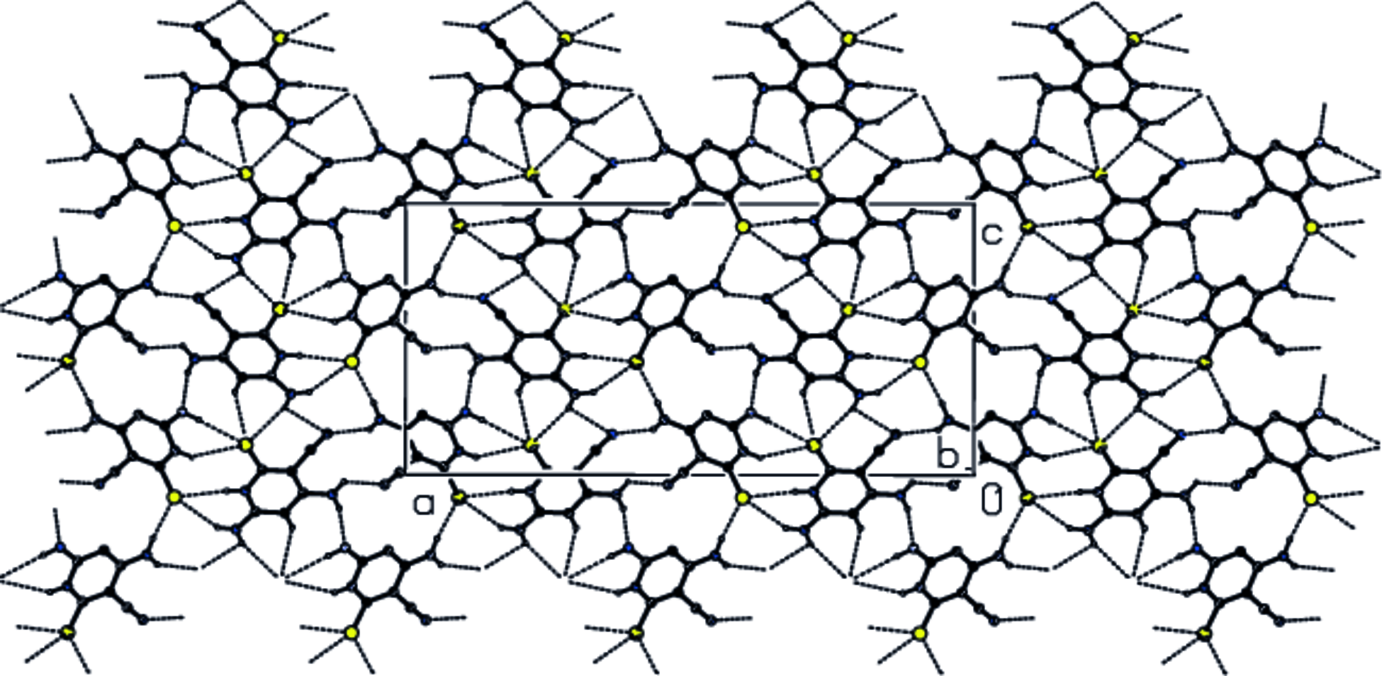

Supplement: Supplementary file 5 [file e-70-0o993-fig2.tif]
